# Ti_2_CT_x_ MXene as a Saturable Absorber for Passively Q-Switched Solid-State Lasers

**DOI:** 10.3390/polym13020247

**Published:** 2021-01-13

**Authors:** Hongfu Huang, Jianwen Wang, Ning Xu, Shunxiang Liu, Guowen Liang, Qiao Wen

**Affiliations:** Key Laboratory of Optoelectronic Devices and Systems of Ministry of Education and Guangdong Province, College of Physics and Optoelectronic Engineering, Shenzhen University, Shenzhen 518000, China; 1900453012@email.szu.edu.cn (H.H.); 1810285062@email.szu.edu.cn (J.W.); 1810285032@email.szu.edu.cn (N.X.); 2150190124@email.szu.edu.cn (S.L.); guowen.liang@polyu.edu.hk (G.L.)

**Keywords:** Ti_2_CT_x_ MXene, saturable absorber, solid-state laser, Q-switched laser

## Abstract

In this work, we successfully fabricated a transmissive saturable absorber (SA) with Ti_2_CT_x_ MXene using the spin-coating method. By inserting the Ti_2_CT_x_ saturable absorber into the diode-pumped solid-state (DPSS) Nd:YAG laser, a stable passively Q-switched operation was obtained near 1.06 μm. At a pump power of 4.5 W, we obtained the shortest pulse duration of 163 ns with a repetition rate of 260 kHz. The corresponding single pulse energy and peak pulse power were 3.638 μJ and 22.3 W, respectively. The slope efficiency and the optical conversion efficiency of the laser were 21% and 25.5%, respectively. To the best of our knowledge, this is the first time that Ti_2_CT_x_ was used in the passively Q-switched solid-state lasers. This work demonstrates that Ti_2_CT_x_ can be a promising saturable absorber for solid-state laser pulse generation.

## 1. Introduction

MXene (a novel class of 2D transition metal carbides and nitrides) is a relatively new material [1,2] that has becomes increasingly attractive for excellent characters, including high optical modulation depth [3], excellent conductivity [4], and good physical stability at room temperature [5]. MXene represents a material system composed of M_n+1_X_n_T_x_ elements, wherein M refers to a transition metal element, X refers to C or N, T refers to the terminations on the surface of the 2D material, and n = 1, 2, 3. Since the first discovery of MXene by Y. Gogotsi in 2011 [1], MXene has been implemented in various fields such as energy storage, electrochemistry, biocatalysts, biochemical sensing, and nonlinear optics. For an instant, Ti_3_C_2_T_x_/CNTs was successfully used as a separator in Li-S battery for the realization of high-performance Li-S batteries [6]. Spraying Ti_3_C_2_T_x_ MXene on commercial polypropylene is a simple, convenient, and effective way to improve the electrochemical performance of Ni-rich cathode [7]. A new biosensor using Ti_3_C_2_T_x_ MXene nanocrystals showed high sensitivity [8]. The MXene has also been considered as a surface plasmon resonance refractive index sensor for biochemical sensing applications [9]. Due to the small average band gap (smaller than 0.2 eV), MXene has the potential to be used in ultrafast photonics as a saturable absorber (SA) [10].

Solid-state laser systems that produce short pulse duration and high pulse energy are required for various applications in medical surgery [11], remote sensing [12], scientific research [13], and laser material processing [14]. Compared with other Q-switched lasers, passively Q-switched diode-pumped solid-state (DPSS) lasers is a simple, cost-effective device to obtain high-peak-power pulses in the nanosecond and sub-nanosecond regimes [15]. For the most part, saturable absorbers (SAs) play a significant role in the realization of passively Q-switched laser pulses. Some kinds of traditional SAs have been developed for many years, including semiconductor saturable absorber mirrors (SESAMs) [16] and transition element (Cr^4+^, V^3+^) doped crystals [17,18]. However, some inherent disadvantages of them limit their applications. The former is expensive and difficult to manufacture, while the latter has not only a narrow operation wavelength band but a high pumping threshold [19]. In recent years, two-dimensional (2D) materials have been used more and more in the field of Q-switched laser pulse generation due to their advantage of compactness, low-cost, and convenience [20,21,22,23,24,25,26,27,28].

Due to the excellent nonlinear optical absorption characteristic, a few SAs based on MXene (Ti_3_C_2_T_x_ [29,30,31,32], Ti_3_CNT_x_ [33], V_2_CT_x_ [34]) have been studied for Q-switched and mode-locked lasers in the past few years. Lately, passively Q-switched solid-state lasers based on Ti_4_N_3_T_x_-SAs were realized in the mid-infrared wavelength region [35]. The few-layered hybrid Ti_3_C_2_(OH)_2_/Ti_3_C_2_F_2_ were used as SAs to make a passively Q-switched Nd:YVO_4_ laser at 1 μm and 1.3 μm [36]. As a member of MXene, Titanium carbide (Ti_2_CT_x_) has only a few relevant reports on its saturable absorption characteristics and its application in passively modulated lasers. Until now, Ti_2_CT_x_ has been successfully used as SA in the fiber laser field. The excellent nonlinear absorption performance of Ti_2_CT_x_ in the mid-infrared field was found by Yi et al. [37]. Our group has previously investigated the optical characteristics of few-layer Ti_2_CT_x_ (T=O, OH, or F) nanosheets (the band gap of the Ti_2_CT_x_ nanosheets is 0.12 eV) and applied them as SAs to obtain ultrafast fiber lasers [38]. Comparing with fiber lasers, solid-state lasers are more suitable for producing high energy short pulse due to their less nonlinear pulse splitting [39]. However, the saturable absorption behavior of MXene Ti_2_CT_x_-SAs near 1 μm applied in DPSS laser has been rarely reported.

In this work, the Ti_2_CT_x_ nanosheets were characterized for their linear and nonlinear absorption properties in the near-infrared band. One Ti_2_CT_x_ transmissive SA was fabricated by spreading the few-layer Ti_2_CT_x_ dispersions over a quartz substrate on a rotary platform. After inserting the Ti_2_CT_x_ SA into the designed plane–concave cavity, passively Q-switched Nd:YAG laser operation near 1 μm was realized. The passively Q-switched Nd:YAG laser generated pulses with a minimum duration of 163 ns, a maximum pulse repetition rate of 260 kHz, a maximum single-pulse energy of 3.638 μJ, and a maximum peak power of 22.3 W.

## 2. Experimental

### 2.1. Fabrication of Ti_2_CT_x_-SAs

**Figure 1:**
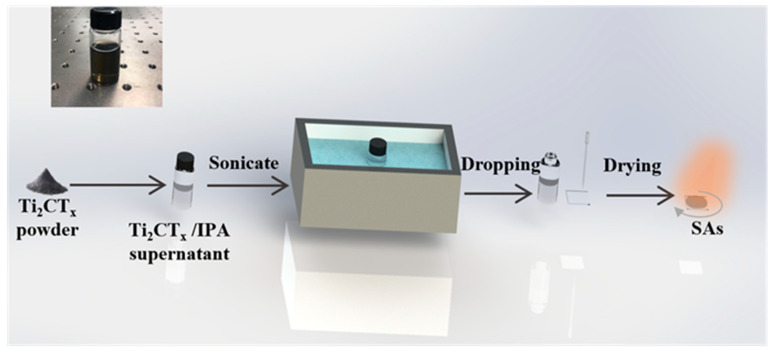
The fabrication process of the transmissive Ti_2_CT_x_-SAs. Insite: the prepared supernatant in IPA.

Commercially available multilayer Ti_2_CT_x_ nanosheet powder (11 technology co., LTD., Changchun, Jilin, China) was used as the starting material. After etching the MAX parent phases of Ti_2_AlC powders in the hydrofluoric acid (HF), the multilayer Ti_2_CT_x_ nanosheets were successfully synthesized. The obtained multilayer Ti_2_CT_x_ nanosheet powder was dissolved into isopropyl alcohol (IPA) and sonicated for 12 h. After the ultrasonic process, the dispersed solution was centrifuged at a speed of 8000 rpm for 30 min. Then the supernatant was collected into a glass bottle and sonicated for 10 min. Figure 1 (Left inside) shows the image of the prepared supernatant in IPA. Then, the prepared solution was dropped on one 25 mm × 25 mm × 1 mm quartz glass sheet (ultrasonicated with alcohol for 10 min) to make a transmissive SAs. The detailed fabrication process of the transmissive Ti_2_CT_x_-SAs by using the spin-coating method is shown in Figure 1.

### 2.2. Characterization of Ti_2_CT_x_-SAs

**Figure 2:**
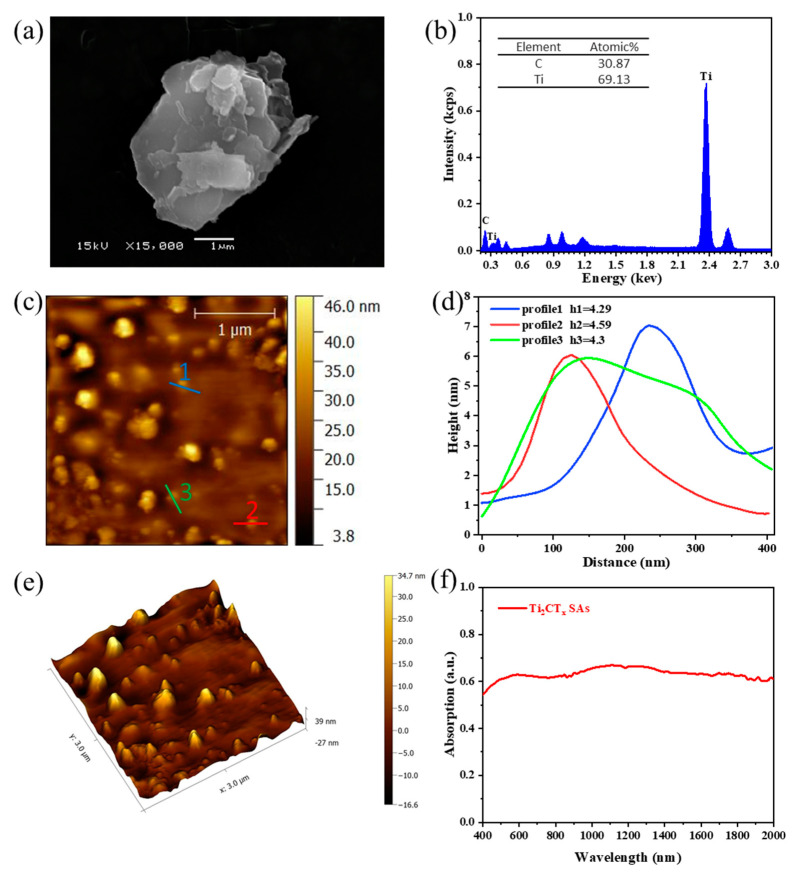
(**a**) SEM image of Ti_2_CT_x_. (**b**) EDS of Ti_2_CT_x_. Left inset: The table of detailed element content distribution. (**c**) AFM image of Ti_2_CT_x_. (**d**) The corresponding thickness of the synthesized Ti_2_CT_x_. (**e**) The 3D topographical image of the Ti_2_CT_x_. (**f**) UV-NIR absorption spectrum of Ti_2_CT_x_ in IPA.

The surface structure of the prepared Ti_2_CT_x_-SAs was observed by using scanning electron microscopy (SEM, JSM-5910LV, JEOL, Tokyo, Japan). As shown in Figure 2a, a clear layered structure can be seen, indicating successful exfoliation. Figure 2b shows the atomic components and corresponding ratio of the as-prepared Ti_2_CT_x_ films measured by the energy dispersive spectrometer (EDS) (Oxford Instruments, Oxford, UK). The percentage of C and Ti are 30.87%, 69.13% in the Ti_2_CT_x_, the corresponding ratio of C to Ti is about 1:2. The distribution of Ti_2_CT_x_ with a few layers and surface morphology thickness was obtained by using the atomic force microscope (AFM, MFP-3D Infinity, Asylum Research, Oxford, UK). According to the measurement, a morphology image is presented in Figure 2c in a square region with dimensions of 3 μm × 3 μm. Three different sections were chosen to determine the thickness of Ti_2_CT_x_ nanosheet. As illustrated in Figure 2d, the corresponding thickness of the synthesized Ti_2_CT_x_ nanosheet was between 4.29 nm and 4.59 nm through the thickness profile. According to previous reports [40,41], the thickness of single-layer Ti_2_CT_x_ is about 1.5 nm, so the number of nanosheet layers in the Ti_2_CT_x_ films is about 3. Figure 2e shows the 3D morphology image of the Ti_2_CT_x_ films, and we can see that the nanosheets are evenly distributed from here. Figure 2f shows the absorption spectra of the Ti_2_CT_x_-SAs in the wavelength range from 400 to 2000 nm measured by a UV/VIS/NIR spectrometer(LAMBDA, Pekin Elmer Inc., Waltham, MA, USA), indicating that the absorption of the sample was measured to be about 66% at 1.0 μm (the absorption of the glass sheet has been taken into account).

**Figure 3:**
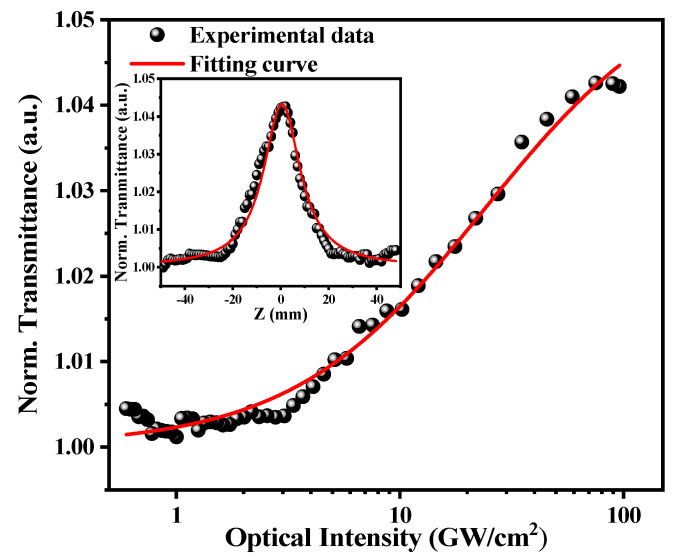
Nonlinear transmission property versus the optical intensity. Inset: the Z-scan curve of the Ti_2_CT_x_-SAs.

One open aperture Z-scan measuring method was used to study the nonlinear characteristics of Ti_2_CT_x_-SAs. Nonlinear optical properties of 2D materials can be affected by the temperature [42,43]. During the experiment, the operating temperature was kept at room temperature (290–300 K). The normalized transmittance in 1064 nm of this material was measured by a commercial Ti: sapphire oscillator (808 nm, 100 fs, 1 kHz,) and an optical parametric amplifier (OPA) system, as shown in Figure 3. The measured data were fitted by Equation (1):

(1) $$T\left(I\right)\;=\;1\;-\;{\Delta T}_{exp}\left(-\frac{I}{I_{sat}}\right)\;-\;T_{ns}$$

where ΔT is the modulation depth, $I_{sat}$ is the saturation intensity, and $T_{ns}$ is the non-saturable loss. By fitting the data with the above equation, we can know the modulation depth and the saturation intensity of Ti_2_CT_x_-SAs are about 4.5% and 32.02 GW/cm^2^, respectively.

### 2.3. Q-Switched Solid-State Lasers with Ti_2_CT_x_-SAs

**Figure 4:**
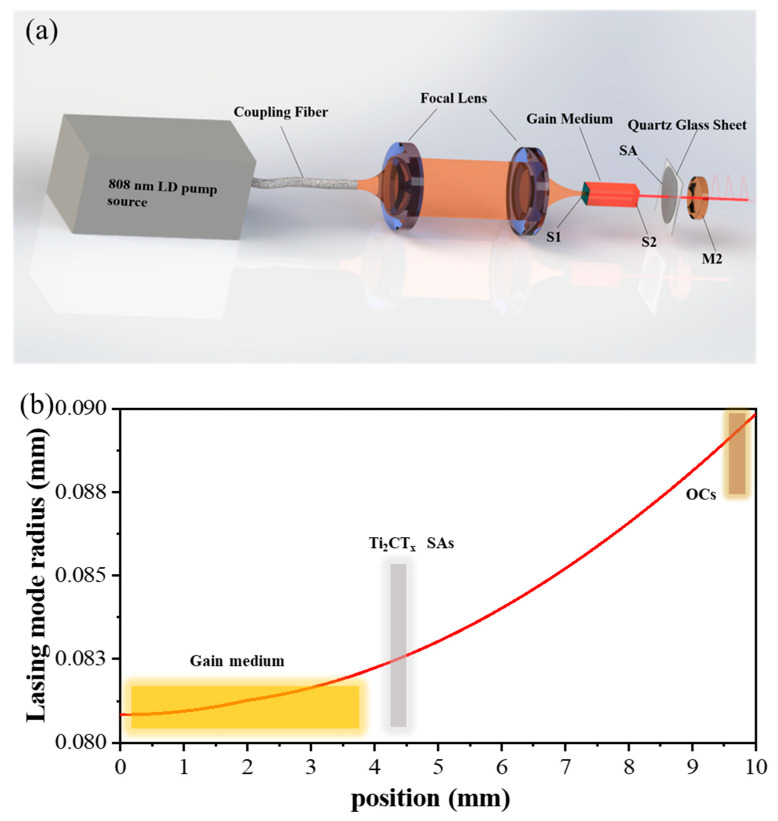
(**a**) Experimental setup for the MXeneTi_2_CT_x_-SAs based passively Q-switched laser. (**b**) The lasing mode radius of the spot in the laser cavity by theoretical estimation.

A single mirror system is specially designed to achieve Q-switched operation with Ti_2_CT_x_- SAs, and the experimental setup is shown in Figure 4a. The pump source is a commercially available fiber-coupled 808 nm diode laser with the maximum output power of 30 W. The fiber core diameter is 200 μm and the NA is 0.22. A collimating focus lens set (1:0.8) is used to focus the pump beam into the laser gain medium with a diameter of 180 μm. The laser gain medium is a 3 mm × 3 mm × 4 mm Nd:YAG (1.2 at%, cut in the [111] direction). The Nd:YAG crystal is wrapped by an indium foil and mounted in a copper block cooled by the circulating water with a temperature of 17 °C. The S1 film (high transmission for 808 nm and high reflection for 1064 nm) plated on the end surfaces of the crystal acts as an input mirror. Another S2 film (high transmission at 1064 nm and high reflection at 808 nm) plays an important role to ensure that the pump light can be absorbed twice by the crystal and, also, to prevent the pump light from affecting the subsequent SAs. The concave output coupler (OC) M2 with a radius of 50 mm was coated with a partial transmission of 5% at 1064 nm. The film S1 and the OC M2 constitute a simple plane-concave resonator, of which the total length was optimized to be about 10 mm. The ABCD matrix method is a matrix (2 × 2) that describes the role of an optical element in the transmission (free uniform medium, thin lens, and mirrors) of a laser beam [44]. The components in the resonant cavity can be regarded as a matrix (2 × 2), and by calculating the matrix, we can get the matrix parameters of the laser at any point in the resonant cavity. The gain medium and the film S1 are equivalent to an optical thin lens and a plane mirror. By the ABCD matrix method, the spot size of each position of the central axis of the resonator can be calculated. The simulated laser beam radius within the resonator is shown in Figure 4b. The Ti_2_CT_x_-SAs was placed 1 mm apart from the gain medium to obtain high intra-cavity optical intensity and the corresponding incident spot radius was estimated at 0.083 mm on the Ti_2_CT_x_-SAs.

## 3. Results and Discussion

**Figure 5:**
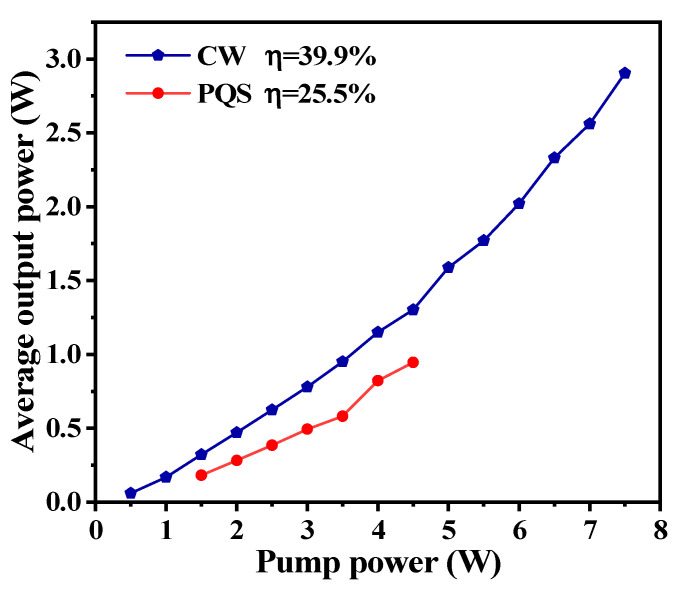
Output power versus pump power at CW (blue dots) and passively Q-switched (PQS) mode (red dots).

Before carrying out the passively Q-switched laser experiment, the continuous-wave (CW) laser operation was investigated first. The output power was measured with a power meter (30A-P-17, Ophir Optronics Solutions Ltd., Jerusalem, Israel). As shown in Figure 5, the threshold pump power was 0.5 W. A maximum CW output power of 2.903 W was obtained at the pump power of 7.5 W, and the corresponding optical conversion efficiency and slope efficiency were 38.7% and 39.9%. By inserting the Ti_2_CT_x_-SAs into the laser cavity, a stable passively Q-switched (PQS) pulse laser was achieved when the pump power was increased at 1.5 W. At the pump power of 4.5 W, a maximum PQS laser average output power of 0.946 W was obtained, corresponding to an optical conversion of 21%, and a slope efficiency of 25.5%. When the pump power exceeded 4.5 W, the passively Q-switched pulse laser became unstable and disappeared. To protect the Ti_2_CT_x_-SAs, experiments were carried out when the power was less than 4.5 W.

**Figure 6:**
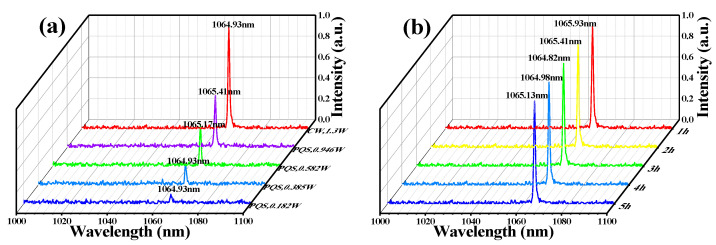
(**a**) Output spectrums of the Nd:YAG CW and PQS laser. (**b**) Output spectrums of the Nd:YAG PQS laser lasted for 5 h at the output power of 0.946 W.

By using a spectrometer (USB4000-VIS-NIR, Ocean Optics Inc., Dunedin, FL, USA) with a range of measurements from 200–1100 nm, the output spectra of CW and PQS Nd:YAG laser were achieved. As shown in Figure 6a, an output wavelength with a center wavelength of 1064.93 nm was acquired at the CW average output power of 1.3 W. While in the PQS mode, the output wavelengths with center wavelengths of 1064.93 nm, 1064.93 nm, 1065.17 nm, and 1065.41 nm were achieved with average output power increasing from 0.182 W to 0.946 W, which means the insertion of Ti_2_CT_x_-SAs rarely affected the central emission wavelength of the Nd:YAG laser. As shown in Figure 6b, the output spectrums of the laser at hourly intervals for five hours of PQS operation (output power: 0.946 W) was measured. And the results show slight changes in the PQS laser output spectrums.

**Figure 7:**
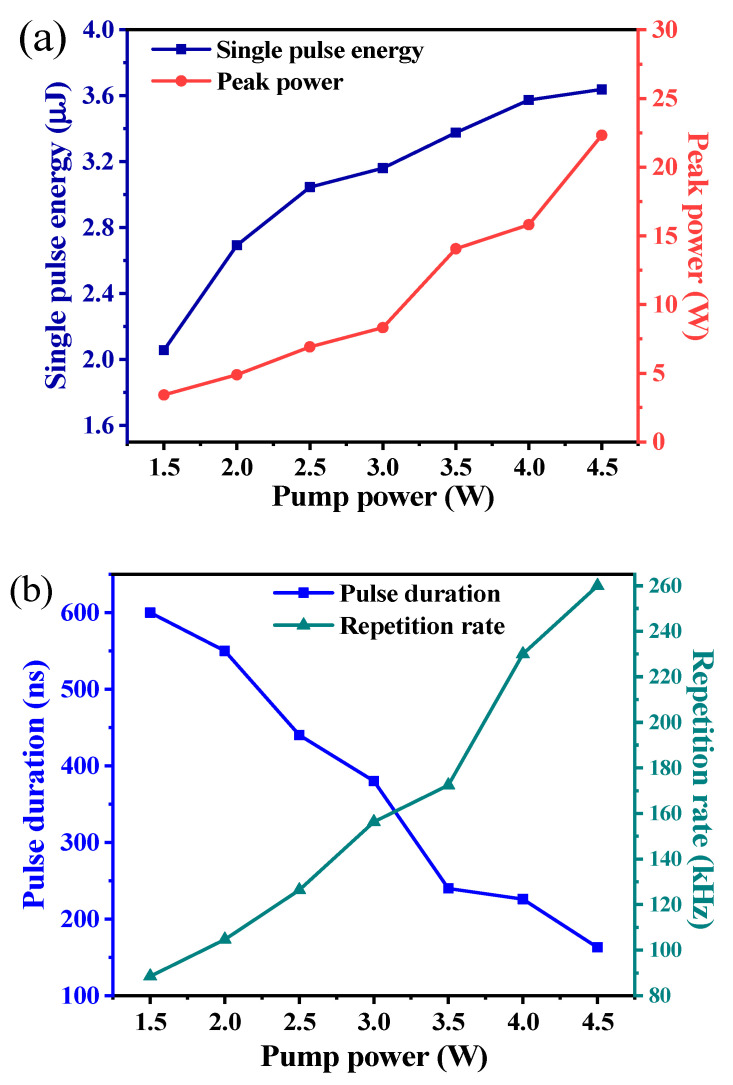
(**a**) Single pulse energy and peak power versus pump power. (**b**) Pulse duration and repetition rate versus the pump power.

**Figure 8:**
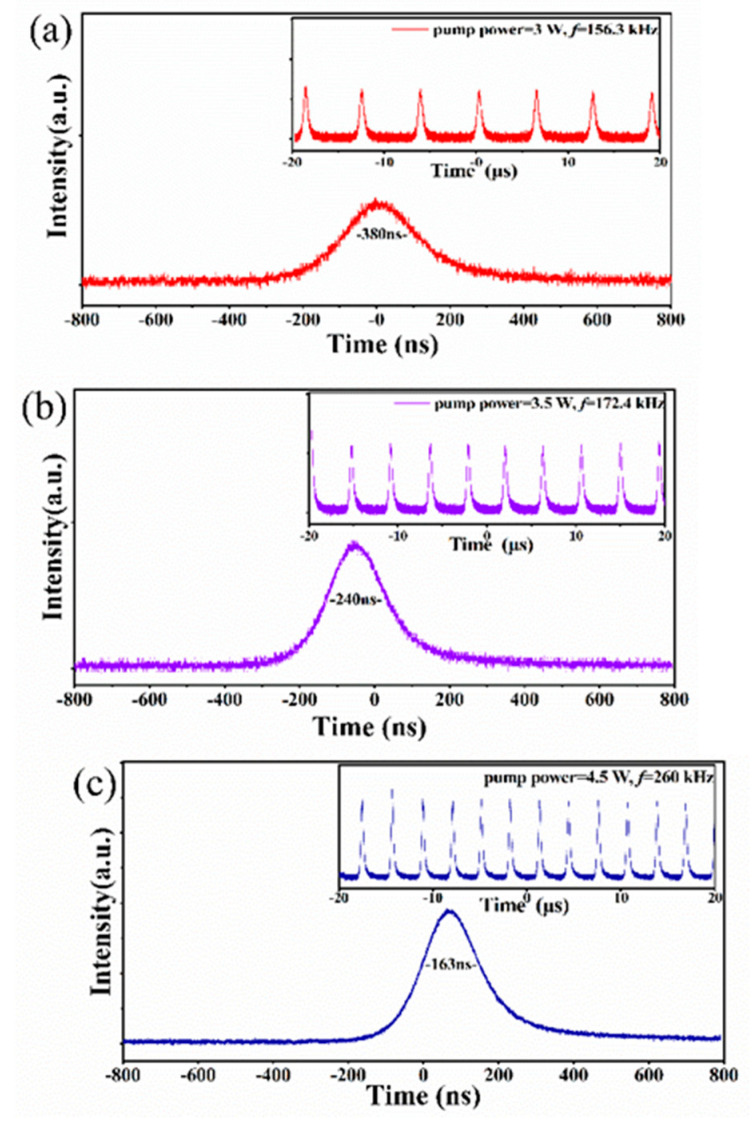
Single-pulse shape (Inset: temporal pulse trains) of the Q-switched lasers at the pump power of 3 W (**a**), 3.5 W (**b**), and 4.5 W (**c**).

One InAsSb (DET10A/M, Thorlabs, Inc., Newton, NJ, USA) photodetector was used to collect the laser output pulse train signal, and a high-speed oscilloscope (DPO4104B, Tektronix, Inc., Shawnee Mission, KS, USA) with a bandwidth of 1 GHz and a sampling rate of 5 GHz is used for display measurement. By inserting the Ti_2_CT_x_-SAs in the cavity and gradually increasing the pump power, the pulse changed from a disordered waveform to a stable Q-switched sequence waveform. Figure 7b shows the pulse duration and repetition rate versus pump power. The pulse duration decreased from 600 ns to 163 ns with the increase of pump power, while the pulse repetition rate increased from 126.5 kHz to 260 kHz. At the pump power of 4.5 W, the shortest pulse duration of 163 ns was obtained. Figure 7a shows the calculated single pulse energy and peak power versus the pump power. The maximum single pulse energy was calculated as 3.638 μJ, corresponding to a maximum pulse peak power of 22.3 W. Figure 8 displays three single pulse shapes and corresponding pulse trains of the Nd:YAG/Ti_2_CT_x_-SAs laser at the pump power of 3 W, 3.5 W, and 4.5 W, respectively, which looks uniform and stable. At the same condition, the pulse train became denser (the pulse repetition rate become higher) with the increasing pump power. The shapes of the pulses were symmetrical, and the pulse duration became shorter with the increasement of the pump power. As the experiment went on, the output pulse train changed slightly (e.g., pulse width, repetition frequency), but the output was still a conventional Q-switched pulse train. The instabilities (output power, rms) of the Nd:YAG/Ti_2_CT_x_-SAs PQS laser was measured to be 3.125% at 0.5 h.

**Figure 9:**
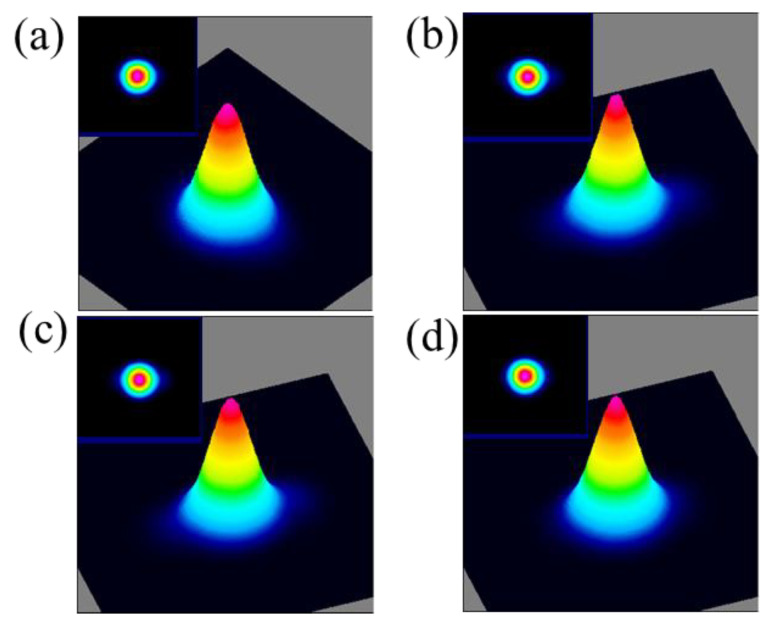
The 2D and 3D spatial power distribution of CW (**a**) and PQS Nd:YAG laser at the pump power of 3 W (**b**), 3.5 W (**c**), and 4.5 W (**d**).

With a laser beam profiling system (BeamGate, Ophir-Spiricon, North Logan, UT, USA), the beam quality of the CW and PQS Nd:YAG laser was evaluated. Figure 9a shows the 2D and 3D spatial power distribution of CW Nd:YAG laser at the pump power of 3 W. Figure 9b–d show the 2D and 3D spatial power distribution of PQS Nd:YAG laser at the pump power of 3 W, 3.5 W, and 4.5 W. These results indicated that the output transverse modes of the CW and PQS Nd:YAG laser are all TEM_00_ modes, which means the out laser beam have a high quality.

The output properties of Q-switched solid-state lasers modulated by different 2D Materials-SAs are shown in Table 1. The Nd:YAG/Ti_2_CT_x_-SAs laser has a shorter pulse duration than the most 2D Materials-SAs based lasers. Compared to other MXene-SAs based lasers, the average output power, output pulse energy, and pulse peak power of the Nd:YAG/Ti_2_CT_x_-SAs laser are much higher. Besides, compared to the Nd:YAG/Ti_3_C_2_T_x_-SAs laser, the average output power, output pulse energy, and pulse peak power are increased by about 10 times, 6 times, and 11 times. The optical conversion efficiency of this Nd:YAG/Ti_2_CT_x_-SAs laser also stands out from the list. The specially designed laser resonator (relatively short cavity, optimal OC) also contributed to the good output characteristics [45,46]. Some MXene/polymers SAs have been successfully used in ultrafast lasers [47,48,49]; by combining MXene with the polymers, the properties of the MXene-SAs have been improved. Further work could be done to improve the properties of the MXene Ti_2_CT_x_-SAs in Q-switched solid-state lasers.

## 4. Conclusions

In summary, based on the nonlinear optical effect of two-dimensional material Ti_2_CT_x_, we applied the few-layer Ti_2_CT_x_ as a new saturable absorber in Nd:YAG solid-state laser to obtain a stable Q-switched pulse output with a central wavelength of 1064 nm. By increasing the pump power from 1.5 W to 4.5 W, the pulse duration decreased from 600 ns to 163 ns, while the corresponding repetition rate, single pulse energy, and pulse peak power are all increasing (90–260 kHz, 2.0−3.638 μJ, 3–22.3 W). With the nonlinear optical properties (ΔT: ~4.5%, *I_sat_*: ~32.02 GW/cm^2^), the Ti_2_CT_x_ two-dimensional material is expected to be a new saturable absorber for generating stable solid-state Q-switched lasers in 1.06 μm.

## Figures and Tables

**Table 1:** Comparison of different 2D materials-SAs used in Q-switched solid-state lasers.

SAs		Λ (nm)	Laser Type	Τ (ns)	F (kHz)	P (mW)	E_SP_ (μJ)	P_P_ (W)	*ε*	Ref.
Graphene		1064	Nd:GdVO_4_	104	600	1220	2.03	19.52	12.2%	[50]
Black phosphorus		1064	Nd:GdVO_4_	495	312	22	0.07	0.141	3.7%	[51]
TIs	Bi_2_Te_3_	1064	Nd:YVO_4_	2000	151.5	170	1.122	0.561	3.2%	[52]
	Bi_2_Se_3_	1064	Nd:GdVO_4_	666	547	32	0.058	0.087	1.7%	[53]
TMDs	ReS_2_	1064	Nd:YAG	139	644	120	0.186	1.34	12.6%	[54]
	MoS_2_	1064	Nd:GdVO_4_	970	732	227	0.31	0.32	8.3%	[55]
MXene	Ti_3_C_2_T_x_	639	Pr:LiYF_4_	264	163	150	0.92	3.48	6.0%	[56]
		1049	Nd:SRA	346	201	130	0.65	1.87	4.6%	[57]
		1064	Nd:YAG (ceramic)	359	186	94.8	0.66	2.04	2.3%	[58]
		1300	Nd:YVO_4_	454	162	30	0.2	0.406	0.6%	[59]
		2100	Ho:YLF	837	35.5	341	20.8	7.43	13.3%	[60]
		2730	Er:CaF_2_-SrF_2_	814	45.3	286	6.32	7.76	10.6%	[61]
		2950	Ho, Pr:LLF	266.7	83.24	105	1.26	4.73	4.5%	[62]
	Mo_2_CT_x_	1064	Nd:YAG	136	261	547	2.09	15.41	9.7%	[63]
		1342	Nd:YVO_4_	222	236	236	1.41	6.36	3.1%	[63]
	Ti_4_N_3_T_x_	2850	Er:Lu_2_O_3_	278.4	113.7	778	6.84	24.57	10.5%	[34]
	hybrid Ti_3_C_2_(OH)_2_/ Ti_3_C_2_F_2_	1064	Nd:YVO_4_	130	508	620	0.6	4.35	12.2%	[35]
		1340	Nd:YVO_4_	390	195	480	2.45	6.25	18.1%	[35]
	Ti_2_CT_x_	1064	Nd:YAG	163	260	946	3.638	22.32	21%	This work

λ, wavelength; τ, pulse duration; F, repetition frequency; P, average output power; E_SP_, single pulse energy; P_P_, peak power; *ε*, optical conversion efficiency; TIs, Topological Insulators; TMDs, Transition-metal sulfides.

## Data Availability

The data presented in this study are available on request from the corresponding author.
